# Primary Biliary Cirrhosis in a genetically homogeneous population: Disease associations and familial occurrence rates

**DOI:** 10.1186/1471-230X-12-110

**Published:** 2012-08-16

**Authors:** Aikaterini Mantaka, Mairi Koulentaki, Gregory Chlouverakis, Jean Marie Enele-Melono, Aikaterini Darivianaki, Maria Tzardi, Elias A Kouroumalis

**Affiliations:** 1 Department of Gastroenterology and Hepatology, University Hospital of Heraklion, P.O. BOX 1352, Heraklion, 71100, Crete, Greece; 2 Department of Social Medicine, Faculty of Medicine, University of Crete, Heraklion, 71100, Greece; 3 Department of Clinical Immunology, University Hospital of Heraklion, PO BOX 1352, Heraklion, 71100, Crete, Greece; 4 Department of Pathology, University Hospital of Heraklion, PO BOX 1352, Heraklion, 71100, Crete, Greece

**Keywords:** Familial pbc, risk factors, cholecystectomy, dyslipidaemia, cancer, educational level

## Abstract

**Background:**

Primary biliary cirrhosis (PBC) is a disease with genetic and environmental pathogenetic background. Chemicals, infectious agents, hormone therapy, reproductive history and surgical interventions have been implicated in the induction of PBC. Familial PBC has been documented in first degree relatives (FDR). Most cohort studies are genetically heterogeneous. Our study aimed to determine eventual lifestyle or disease associations and familial occurrence rates in a genetically homogeneous and geographically defined population of PBC patients.

**Methods:**

111 consenting PBC patients, were compared with 115 FDR and 149 controls matched for age, sex, Cretan origin and residence. All participants completed a questionnaire regarding demographics, lifestyle, medical, surgical and reproductive history. Significant variables on the univariate analysis were analyzed by multivariate analysis using a forward step-wise logistic regression model.

**Results:**

Dyslipidaemia was found in 69.4% of patients, 60% of FDR and 40.9% of controls (p < 0.0001 and p = 0.003 respectively), autoimmune diseases in 36.9% of patients, 30.4% of FDR and 13.4% of controls (p < 0.0001 and p = 0.011 respectively). Hashimoto’s disease (p = 0.003), Raynaud syndrome (p = 0.023) and Sjögren syndrome (p = 0.044) were significantly associated with PBC. On multivariate analysis statistically significant associations were found with primary educational level (AOR 2.304, 95% CI 1.024-5.181), cholecystectomy (AOR 2.927, 95% CI 1.347-6.362) and the presence of at least another autoimmune disease (AOR 3.318, 95% CI 1.177-6.22). Cancer history was more frequent in patients than in controls (p = 0.033). Familial PBC was found to be 9.9%.

**Conclusions:**

Dyslipidaemia and autoimmune diseases were significantly increased not only in patients as expected but also in their FDR. An increased prevalence of malignancies was found in patients. Primary educational level, cholecystectomy and the presence of at least another autoimmune disease were found as putative risk factors for PBC. No association was found with smoking, urinary tract infection or reproductive history. The reported high familial occurrence of PBC could imply screening with AMA of FDR with at least another autoimmune disease.

## Background

Primary biliary cirrhosis (PBC) is a cholestatic disease of unknown aetiology primarily affecting middle-aged women. It is characterized by progressive destruction of the small intrahepatic bile ducts that leads to ductopenia, fibrosis and ultimately liver cirrhosis. The serologic hallmark of PBC in 95-98% of the patients are the M2 anti-mitochondrial autoantibodies (AMA), directed against the E2 sub-unit of the pyruvate dehydrogenase multi-enzyme complex (PDC) located in the inner membrane of the mitochondria. [1] It is considered an autoimmune disease resulting from the interaction of multiple environmental factors, the immune system and the liver of genetically susceptible individuals [2].

The role of the genetic factors in PBC is strongly supported by the 63% concordance rate in monozygotic twins, the second highest reported in autoimmunity after celiac disease [3]; as well as the high familial PBC occurrence rates reported 4% [4] to 9% [5] in first-degree relatives (FDR) of PBC patients [6]. Moreover the presence of AMA with no other clinical evidence of disease is reported in 13.1% [5]. The significant coexistence of other autoimmune diseases and/or other autoantibodies in both patients and their FDR, also indicates the genetic background of PBC [7].

Geographic clusters of the disease have been found in small areas, a fact that underlines the significance of environmental factors in the induction of PBC [8-11]. A number of microorganisms (E. Coli [12], Mycobacteria [13], Novosphingovium aromaticivorans [14,15], Lactobacillus species [16], Chlamydia pneumoniae [17,18], Helicobacter pylori [19]), viral infections (human beta retrovirus [20], MMTV [21]), xenobiotics [10,22,23] and halogenated compounds found in hair dyes, nail polish and cigarette smoke [24,25] have been associated with PBC as environmental factors.

The age at first pregnancy, the frequency of pregnancies, abortions, hormone replacement therapy (HRT) and a previous history of obstetric cholestasis, as well as urinary tract infections (UTI), tonsillectomy, appendectomy and cholecystectomy have also been reported as possible risk factors [4,24-26].

Many studies include patients with ethnic variations that may influence the results. Studies in populations that share the same genetic background, common environment and low migration rates make it more plausible to identify environmental and/or genetic factors playing a role in a disease’s induction.

Therefore we analyzed the demographics, lifestyle, medical and surgical history in a genetically and ethnically homogeneous group of Cretan PBC patients and their FDR compared with a control group matched for age, gender and residence with the patients in order to identify familial occurrence rates, associated lifestyle factors and/or comorbidities.

## Methods

Between March and October 2007 we mailed study invitation letters to 196 PBC patients and their FDR (parents and children), who were regularly followed at the Department of Gastroenterology and Hepatology of the University Hospital of Heraklion (Crete, Greece), referral centre for liver disease in the island. The letter explained in detail the scientific data concerning the genetic and environmental factors in the pathogenesis of the disease, and pointed out the goals and the procedures of the study for both patients and their FDR. A hundred-eleven patients consented (56.6%), 40 patients declined and 45 patients did not respond. A hundred-fifteen FDR (75 females, 40 males) also consented to be enrolled (55% of the living FDR). A hundred-forty-nine unrelated controls matched to PBC patient by age (±2.5 years), gender, Cretan origin and residence were also enrolled at the study. The unrelated control group was enrolled among the visitors of the hospital.

All study participants, after signing an informed consent, completed a questionnaire through an interview performed by the same doctor. The questionnaire included information regarding demographics (age, gender, place of origin and residence), socioeconomics (profession, educational status), lifestyle (body mass index (BMI) grouped as ‘high’ (>30), ‘medium’ (25–30), and ‘low’(<25), smoking status in 20≤, >20 packs/year including past smoking, weekly alcohol consumption analyzed as “no use” (no consumption), “use” (less than 14 units of alcohol per week for men and 7 units women) or “misuse” (more than 14 units of alcohol per week for men and 7 units for women) counting 1 unit equal to 12gr of alcohol. ‘Hair dye use’ is referred as at least once a month per year, whereas ‘no hair dye use’ as never used. ‘N;ail polish use’ is referred as ≥10 times per year, whereas ‘no nail polish use’ is referred as occasional use or never used. Medical and surgical history questions referred at the period prior to PBC diagnosis. Moreover detailed questions concerning the frequency of vaginal and UTI, thyroid gland dysfunction, chronic diseases (hypertension (HT), diabetes mellitus (DM), dyslipidaemia, coronary artery disease (CAD), peripheral vasculopathy, asthma/chronic obstructive pulmonary disease (COPD)), allergies, other possible liver diseases and other autoimmune diseases were recorded. Diagnosis of Hashimoto’s disease was established by the combination of hypothyroidism and elevated thyroperoxidase (TPO) and thyroglobulin (TG) antibodies. For the female participants a detailed reproductive history prior to PBC diagnosis was assessed, which included the date of menarche, date of first pregnancy, number of pregnancies, childbirths, abortions or miscarriages; menopausal status, use of oral contraceptives or HRT and gynecological surgical history.

Collection of data lasted from January 2008 to December 2010. Medical files of the PBC patients were retrieved and reviewed and data concerning their clinical parameters, liver biopsies, Mayo risk score at diagnosis and recent laboratory tests were annotated. FDR and controls after the interview were also clinically examined and tested for: alanine aminotransferase (ALT), aspartate aminotranferase (AST), alkaline phosphatase (ALP), γ-glutamine transferase (γ-GT), bilirubin, glucose, urea, cholesterol, triglycerides, high density lipoprotein (HDL), low density lipoprotein (LDL), fT3, fT4, TSH, anti-TPO and anti-TG, immunoglobulins IgA, IgG, IgM, rheumatoid factor (RF). Viral hepatitis B and C markers were assessed by ELISA.

Antinuclear antibodies (ANA) were tested by indirect immunofluorescence on Hep-2 substrate with 1/80 cut-off of positivity. Anti-mitochodrial antibodies (AMA) and anti-smooth muscle antibodies (SMA) were tested by an indirect immunofluorescence (IIFL) assay of Nova Lite ^TM^ (IFA) on Mouse Kidney & Stomach substrate (Inova Diagnostics, San Diego CA, Inc) and a titre of ≥1/40 was considered positive, according to the manufacture’ s instructions. Anti-M2 antibodies were assessed by qualitative and quantitative ELISA (AESKULISA, German). Negative was 1-12U/ml, grey zone 12-18U/ml and positive >18U/ml.

The study protocol conforms to the ethical guidelines of the 1975 Declaration of Helsinki (6th revision, 2008) and was approved by the Hospital’s ethical committee.

### Statistical analysis

All data were evaluated in two sets of comparisons: patients with controls, FDR with controls. Comparisons were made by Student’s *T*-test for continuous variables, Fisher’s exact probability test and the *χ*2 test for the analysis of categorical variables. All variables found to be significant in the univariate analyses for PBC patients were entered into the multivariate analyses using a forward step-wise logistic regression model (0.05 for entry and 0.10 for removal probability). A *p*-value of <0.05 was considered statistically significant. Statistical analyses were performed, using the SPSS software package (version 18, SPSS Inc. Chicago, IL, USA).

## Results

The diagnosis of all PBC patients was based on compatible clinical, immunological and histological parameters and all patients were on ursodeoxycholic acid 15 mg/kg since diagnosis. The mean age and the mean Mayo risk score at the time of diagnosis was 56.87 ± 11.42 years and 4.7 ± 1.6, respectively. A hundred and one patients (86 females) were AMA positive in a titre ≥1/40 on IIFL with M2 higher than 18 U/ml on ELISA, while 10/111 (8 females) were AMA negative. Fourty-three of the 111 PBC patients were ANA positive, 16 with MND pattern, 5 MND and peri-nuclear, 4 peri-nuclear, 1 peri-nuclear and anti-nucleolar, 12 speckled, 1 speckled and MND, 1 speckled and anti-nucleolar and 3 diffuse.

Fourteen patients were at stage IV at diagnosis (9 on liver biopsy, and 5 AMA positive with clinical evidence of portal hypertenstion that did not underwent liver biopsy). Of those to the end of the study, two died after diagnosis of HCC, one with HCC is still alive and 3 are alive with de-compensated cirrhosis.

Seventeen more patients were at stage III (2 AMA negative), 3 of those at stage IV alive at the end of the study, one de-compensated. Two dead (one AMA negative) of liver related causes, one within 30 days of OLT.

According to liver biopsy results at diagnosis, 79 (8 AMA negative) PBC patients were at an early stage (Ludwig I-II). During the study period three died of non liver related causes. Five are at stage IV (1 de-compensated) at the end of the study.

One female AMA positive patient refused to undergo biopsy for staging.

Mild piecemeal necrosis was present in 42.35% of the biopsies, moderate in 30.6% and no piece-meal in 27.05%.

Mean follow-up from diagnosis to interview was 91.7 ± 61 months. The mean age and the mean Mayo risk score at 2010 was 64.8 ± 11.9 and 4.99 ± 1.46, respectively.

### Sociodemographic and lifestyle characteristics

The results of the sociodemographic at interview and lifestyle variables of the three study groups are seen in Table 1.

**Table 1:** Demographic, Anthropometric and Lifestyle variables of PBC patients, FDR and Controls

	**Controls N (%)**	**PBC N (%)**	**PBC vs controls p-value**	**FDR N (%)**	**FDR vs controls p-value**
Female sex	127 (85.2%)	94 (84.7%)	NS	75 (65.2%)	NS
Mean age (y)	64.6 ± 11.9	64.8 ± 11.9	NS	43.59 ± 13.7	<0.0001*
Urban residence	96 (64.4%)	58 (52.3%)	NS	89 (77.4%)	0.03*
Rural residence	53 (35.6%)	53 (47.7%)	0.05*	26 (22.6%)	NS
No Education	13 (8.7%)	2 (1.8%)	NS	0	NS
Elementary	53 (35.6%)	61 (55%)	0.01*	14 (12.2%)	<0.0001*
High school	64 (43%)	29 (26.1%)	0.01*	59 (51.3%)	NS
University	19 (12.8%)	19 (17.1%)	NS	42 (36.2%)	<0.0001*
BMI 25-30	71 (47.7%)	44 (39.6%)	NS	32 (27.8%)	0.002*
Mean BMI	27.2 ± 4.25	27.04 ± 4.85	NS	25.58 ± 4.42	0.003*
Active smokers	35/54 (64.8%)	22/36 (61.1%)	NS	48/62 (77.4%)	NS
Past smokers	19/54 (35.2%)	14/36 (38.9%)	NS	14/62 (22.6%)	NS
Non smokers	94 (63.1%)	75 (67.6%)	NS	52/114 (45.6%)	0.006*
Alcohol misuse	9/17 (52.9%)	13/15 (84.6%)	NS	13/34 (38.2%)	NS
Alcohol use	8/17 (47.1%)	2/15 (15.4%)	NS	21/34 (61.8%)	NS
No alcohol use	133 (89.3%)	99 (89.2%)	NS	81 (70.4%)	<0.0001*
Hair Dye use	75 (50.3%)	45 (40.9%)	NS	NA	NA
Nail Polish use	47 (31.5%)	26 (23.6%)	NS	NA	NA

Note: Continuous variables are expressed as mean ± standard deviation, NS: not significant, NA: not available, ^*^Statistically significant.

From the 115 FDR 20% were siblings (8 brothers and 15 sisters), 76.5% were children (56 daughters and 32 sons) and 4 were mothers (3.5%) of PBC patients.

More PBC patients than controls had an elementary education (p = 0.01) and more FDR had a University degree (p < 0.0001). Among lifestyle factors, alcohol consumption and smoking were more frequent in FDR than controls (p < 0.0001 and p = 0.006, respectively), whereas mean BMI was found significantly lower in FDR (p = 0.003).

### Prevalence of Autoimmune diseases in patients and FDR

#### PBC vs Controls

ANA positive were 38.7% vs 16.7% of controls (p = 0.002, OR 3.01 95% CI 1.5-6.02), SMA positive 18.8% vs 10.7% of controls (OR 1.93 95%CI 0.82-4.52), 75.5% of patients vs 9.9% of controls had elevated IgM (p < 0.0001, O.R. 27.8 95%CI 11.9-66.7), with a mean value of IgM = 330.3 ± 227.7 mg/dl vs controls IgM = 109.9 ± 50.7 mg/dl (p < 0.0001and IgM normal range 27-170 mg/dl).

The distribution of autoimmune diseases in PBC patients and controls are shown in Table 2. Among other autoimmune diseases diagnosed in PBC patients were: autoimmune thrombocytopenia in 2/111, multiple sclerosis in 1/111, celiac disease in 1/111, vitiligo in 1/111, autoimmune gastritis in 3/111, Guillain Barre syndrome in 1/111, BOOP in 1/111, vasculitis of autoimmune origin in 1/111, antiphospholipid syndrome in 1/111. Most patients had more than one autoimmune disease. There was a patient who had simultaneously rheumatoid arthritis, Sjögren syndrome, Raynaud syndrome, psoriasis, Hashimoto’s disease and eczema.

**Table 2:** Distribution of autoimmune diseases in PBC patients, controls and FDR

**Autoimmune disease**	**PBC patients (N = 111)**	**Controls (N = 149)**	**PBC vs controls p-value, OR (95% C.I.)**	**FDRs (N = 115)**	**FDR vs controls p-value OR (95%C.I.)**
RA	4.5% (5/111)	0.7% (1/149)	0.087, 7 (0.79-56.64)	3.5% (4/115)	0.171, 5.32 (0.59-47.62)
S.L.E	3.6% (4/111)	1.3% (2/149)	0.407, 2.75 (0.49-15.38)	1.7% (2/115)	1, 1.3 (0.18-9.35)
Scleroderma	1.8% (2/111)	0% (0/149)	0.181, 1.02 (0.99-1.04)	0.9% (1/115)	0.436, 1 (0.99-1.03)
Sarcoidosis	2.7% (3/111)	0.7% (1/149)	0.316, 4.11 (0.42-40)	0.9% (1/115)	1, 1.3 (0.08-20.83)
Sjögren Syndrome	5.4% (6/111)	0.7% (1/149)	0.044*, 8.47 (1–71.43)	0% (0/115)	1, 0.99 (0.98-1.01)
Raynaud	6.3% (7/111)	0.7% (1/149)	0.023*, 10 (1.21-83.33)	2.6% (3/115)	0.321, 3.97 (0.41-38.46)
Eczema	3.6% (4/111)	2.7% (4/149)	0.727, 1.35 (0.33-5.55)	2.6% (3/115)	1, 0.97 (0.21-4.42)
Psoriasis	1.8% (2/111)	1.3% (2/149)	1, 1.35 (0.19-9.71)	3.5% (4/115)	0.409, 2.65 (0.48-14.71)
IBD	1.8% (2/111)	0.7% (1/149)	0.577, 2.72 (0.24-30.3)	0% (0/115)	1, 0.99 (0.98-1.01)
Hashimoto	18% (20/111)	6% (9/149)	0.003*, 3.41 (1.49-7.81)	13.9% (16/115)	0.035*, 2.51 (1,07-5,92)
At least another autoimmune disease	36.9% (41/111)	13.4% (20/149)	<0.0001*, 3.77 (2.06-6.94)	26.1% (30/115)	0.011*, 2.28 (1.21-4.27)

OR = Odds Ratio,^*^Statistically significant.

### FDR vs Controls

Four PBC patients were FDR of other patients resulting in a known familial history of 3.6%. Occurrence of PBC was diagnosed in another 7 FDR (6.3%) (Table 3). Five AMA positive among them underwent liver biopsy that confirmed PBC histologically (4 FDR stage I, 1 FDR stage III). The sixth was a 76 year old AMA + sister with elevated cholestatic liver enzymes that did not consent to undergo liver biopsy. An AMA negative, ANA positive 29 year old daughter with elevation of γ-GT, had a liver biopsy compatible with stage I PBC, i.e. Autoimmune Cholangitis. Addition of the 4 already known cases of familial PBC to the 7 new confirmed cases, results in a 9.9% prevalence of familial PBC in Crete. No autoantibodies were identified in the control group.

**Table 3:** Laboratory features of FDR with abnormal LFT

**Index patient**	**Relation**	**Age**	**ALT**	**γGT**	**ALP**	**TBil**	**IgM**	**ANA**	**AMA**	**Autoimmune Diseases**	**Diagnosis of liver disease**
XN	Sister	65	24	34	**161**	0.61	**201**	-	**1:320/M2**	None	PBC
DA	Sister	76	**65**	**527**	**816**	**1.29**	**1360**	-	**1:160/M2**	None	PBC
MD	Mother	55	**90**	**213**	**362**	**4.7**	**303**	**1:80MND**	**1:160/M2**	None	PBC
PK	Sister	67	**49**	**57**	**140**	**1.4**	**296**	**1:80MND**	**1:320/M2**	Sarcoidosis	PBC
BK	Son	43	**57**	39	90	**1.43**	81.9	-	-	None	Liver steatosis
	Daughter	51	17	15	63	0.85	**238**	-	**1:160/M2**	Hashimoto	PBC
D	Mother	80	21	**57**	**111**	0.79	**250**	**1:640**	-	None	Refused f-up
	Brother	50	**56**	**152**	95	0.68	**180**	-	**1:160/M2**	None	PBC
TM	Daughter	29	**89**	33	126	0.3	**193**	**1:80**	-	None	AIC
PE	Daughter	43	16	11	**191**	0.53	**211**	-	-	Hashimoto, MS	Refused f-up
	Son	54	37	36	**156**	0.64	**189**	-	-	Hashimoto	Refused f-up
AT	Daughter	44	27	**55**	68	0.5	132	-	-	None	Nothing found
KA	Brother	54	24	**61**	58	0.41	120	-	-	None	Nothing found
KX	Son	43	**58**	16	56	0.65	35	-	-	None	Nothing found
ZP	Daughter	56	**83**	27	**102**	0.42	114	-	-	RA,HistiocytosisB	Nothing found
KF	Son	25	**62**	27	69	1.1	102	-	-	None	Liver Steatosis
B	Son	38	**72**	**61**	99	0.51	54.2	-	-	None	Liver Steatosis
M	Son	53	17	**56**	55	1.47	-	-	-	None	Alcohol abuse
ST	Brother	34	35	**54**	60	0.48	89.6	-	-	None	Liver steatosis
	Son	22	**74**	16	42	0.5	90.2	-	-	None	Alcohol abuse
L	Son	44	**79**	**87**	84	0.83	**208**	-	-	None	Refused f-up
TX	Son	55	36	44	**188**	0.85	34	-	-	None	Liver Steatosis
Z	Daughter	18	**89**	31	118	0.3	-	-	-	None	EBV infection

Normal values: AST 8-40U/l, ALT 8-40U/l, γGT 10-50U/l, ALP 40-150U/I, Bilirubin 0.1-1.3 mg/dl, IgM 25-170 mg/dl. In bolded numbers are values over the normal range.

30.8% of FDR had elevated IgM levels above 170 mg/dl, mean value 151.6 ± 136.1 mg/dl (range 23-1360 mg/dl) vs 9.9% of the controls, mean IgM value 109.9 ± 50.7, range (24.8-303 mg/dl), p = 0.009. Increased IgG levels above 1545 mg/dl, were also observed in 12.5% of FDR, mean value 1220.8 ± 263.3 compared to 1.2% of controls, mean value 1028.4 ± 266.2, p = 0.004. ANA (23.1% vs 16.7%) and SMA positive autoantibodies did not differ significantly between the two groups (16.7% vs 10.7%) p = 0.285 for both.

The distribution of other than PBC autoimmune diseases in FDR are shown in Table 2. Overall in 35 FDR (30.4%) at least one autoimmune disease (including the PBC) was identified.

### Other associated diseases

#### PBC patients vs Controls

Dyslipidaemia (69.4% vs. 40.9%, p < 0.0001 OR 3.27, 95% CI 1.95-5.49), and thyroid gland dysfunction other than autoimmune thyroiditis (44.1% vs. 26.2%, p = 0.003, OR 2.23, 95% CI 1.32-3.76) were significantly higher in patients, compared to controls. There were significant differences in the mean cholesterol value (224.5 ± 57.7 mg/dl vs 188.1 ± 41.2 mg/dl p < 0.0001), but not in the mean values of triglycerides between patients and controls (126 ± 45 mg/dl vs. 140.1 ± 73.75 mg/dl).

Diagnosis of HT, DM, CAD, peripheral vasculopathy, asthma/COPD and allergies did not differ significantly between the two groups. UTIs did not differ between groups (p = 0.065) and more than 10 UTI/lifetime were reported by 9.7% patients vs. 6.9% of the controls (p = 0.187).

Compared to controls, patients reported more often a history of cancer (8.1% vs. 2%, p = 0.033, OR 4.29 95% CI 1.13-16.13) and the distribution was: 3 HCC, 1 breast cancer, 1 colon cancer, 1 endometrial cancer, 1 gastric MALT lymphoma, 1 basal cell skin carcinoma, 1 of unknown origin vs. 2 breast and 1 pancreatic cancer. Cholecystectomy differed significantly between the two groups (18.9% vs. 7.4%, p = 0.007 OR 2.92 95% CI 1.35-6.37) while tonsillectomy, appendectomy, and thyroid gland surgery did not.

### FDR vs Controls

Abnormal liver enzymes test were found in 23/115 FDR. Their details are seen in Table 3. All relatives were negative for B and C hepatitis viral markers while drug-induced hepatotoxicity was excluded, as they had not used any medicine or herbal substance for the past six months.

Forty two FDR reported the presence of thyroid gland dysfunction that did not differ between FDR and controls (36.5% vs 26.2%, p = 0.081). HT (p < 0.0001, OR, 0.12; 95% CI: 0.058-0.23), DM (p < 0.0001, OR, 0.17; 95% CI: 0.063-0.44) and CAD (p = 0.046, OR, 0.14; 95% CI: 0.02-1.09) were found more often in controls, a fact justified by the age factor. Nonetheless, FDR despite their younger age, had hyperlipidaemia (elevation in total cholesterol and/or triglycerides, or recent treatment with statins) more frequently than controls (60% vs 40.9%, p = 0.003). Peripheral vasculopathy, asthma/COPD and allergies did not differ significantly between the two groups (p = 0.141, p = 0.195 and p = 0.42 respectively). The frequency of aeroallergens, as allergic factor was greater in FDR, while penicillin and b-lactams allergies were reported more often among controls (p = 0.031). Compared to FDR, controls reported significantly higher prevalence of UTI (p = 0.002), kidney stones and cysts (p = 0.038). A daughter and a mother of 2 PBC patients were diagnosed with ovarian and breast cancer respectively. Surgical history including tonsillectomy (14.8% vs 12.1%, p = 0.864, OR, 0.9; 95% CI: 0.46-1.78) appendectomy (11.3% vs 18.8%, p = 0.123, OR, 0.55; 95% CI: 0.27-1.12), cholecystectomy (7% vs 7.4%, p = 1, OR, 0.94; 95% CI: 0.36-2.41) and thyroid gland surgery (7% vs 3.4%, p = 0.252, OR, 2.15; 95% CI: 0.68-6.76) did not differ significantly between FDR and controls.

### Reproductive history of PBC patients, FDR and controls

Results and comparisons among the three groups are shown in Table 4. All parameters with significant differences between FDR and controls can be explained by the difference between the menopausal women in the two groups (i.e. 18 menopausal FDR vs. 108 controls) as expected by the age difference.

**Table 4:** Reproductive history of female PBC patients, FDR and Controls

	**Controls N =127**	**PBC N = 94**	**PBC vs Controls p-value**	**FDR N = 75**	**FDR vs Controls p-value**
Mean age at menarche	13 ± 1.6	13.13 ± 1.4	NS	13.1 ± 1.35	NS
Mean age at last period	49.2 ± 4.6	48.6 ± 4.8	NS	46.6 ± 5.9	0.039*
Mean age at first pregnancy	24.9 ± 4.8	24.3 ± 4.7	NS	23.35 ± 4.7	NS
Mean duration of period	5.1 ± 1.2	5.3 ± 1.6	NS	5.35 ± 1.2	NS
Number of pregnancies	2.8 ± 2	2.9 ± 1.6	NS	2.1 ± 1.9	0.018*
Number of children	2 ± 1.1	2.2 ± 1.1	NS	1.6 ± 1.3	0.009*
Normal child birth	74.8%	84%	NS	61.6%	NS
Caesarean section	15.7%	12.8%	NS	21.9%	NS
Miscarriage	27.6%	29.8%	NS	20.5%	NS
Abortion	12.6%	13.8%	NS	15.1%	NS
HRT	10.2%	16.3%	NS	26%	0.005*
Hysterectomy	10.2%	17%	NS	8.2%	NS
Ovariectomy	14.2%	18.1%	NS	13.7%	NS
Uterine fibroids	20.5%	21.3%	NS	9.6%	0.05*
Vaginal infection	28.3%	23.3%	NS	42.5%	0.045*

* Statistically significant.

### Multivariate analysis

Variables entered in the multivariate analysis for all patients were rural residence, primary educational level, RA, Sjogren syndrome, Raynaud syndrome, Hashimoto, the presence of at least another autoimmune disease, thyroid gland dysfunction, cholecystectomy and cancer. Primary educational level (AOR 2.304, 95% CI 1.024-5.181), cholecystectomy (AOR 2.927, 95% CI 1.347-6.362) and the presence of at least another autoimmune disease (AOR 3.318, 95% CI 1.177-6.22) were a gender- independent risk factor associated with PBC.

## Discussion

In this case control study we report the prevalence of autoimmune and other co morbidities as well as the sociodemographic and lifestyle factors associated with PBC in an ethnically homogeneous and geographically defined group of PBC patients. The definite advantages of population isolates, such as the Cretan population, referring to more uniform environment, genetic homogeneity and low migration rates give an added value at the study.

The limitation of the study, as with all studies that use questionnaires, is that it comprehends the risk of putative reports that lead to bias. We lowered this risk by interviewing all participants by the same doctor and validated the collected data for the PBC patients, by patient record review.

The potentially low percentage of patients that agreed to participate in the study (56.6%), could be justified by Cretan cultural ethics and prejudices, the advanced age at diagnosis, rural residence (almost half of our patients) and their low educational level. The same reasons could also explain the less frequent use of hair dye and nail polish in our patients. Indeed our findings do not support previous findings in other populations indicating nail polish or hair dye [4,25,27] as a putative risk for PBC.

The educational level of our PBC patients was lower than the controls and of that reported in other populations [4,25] and was found to be an independent predictor for the disease. Previous American and French studies reported lower BMI of PBC patients compared with their controls [4,25] but this was not found in the Cretan population. Most patients in this study although not different from controls were overweight. Current or previous smoking did not differ between PBC and controls but the passive exposure to tobacco smoke was not investigated.

Similar prevalence of autoimmune diseases in our controls with those of the American study (13.4% vs 13%) was found. However our patients had an even higher prevalence of at least one autoimmune disease compared to theirs (36.9% vs 32%) [7]. As in previous studies [7,25] the most frequent autoimmune diseases with very high OR in univariate analysis were autoimmune thyroiditis, Raynaud and Sjögren syndrome, all found to be significantly associated with PBC while the presence of at least another autoimmune disease was found to be an independent risk factor for the disease.

FDR also had a significant higher prevalence of autoimmune diseases (30.4%). The most frequent autoimmune disease found in FDR was Hashimoto (13.9%), followed by PBC (9.9%). Indeed the known prevalence of PBC in FDR in Crete prior to the study was 3.6% similar to the 6% reported in the US study, the 5% in UK patients [7], the 4% in France and the 5.1% in Japan [28]. Nonetheless the familial screening of FDR during the study raised the PBC prevalence in FDR to 9.9%, a figure closer to the 9% reported by Lazaridis et al [5].

Although most patients were overweight and with elevated cholesterol, DM, HT, and CAD did not differ between patients and controls in accordance with the hypothesis that PBC patients may be protected against the metabolic syndrome, due to the prevention of LDL oxidation by lipoprotein X, the antioxidant effects of bilirubin and/or the elevated levels of adiponectin [29-31].

Similarly FDR as expected by the age factor had lower prevalence of HT, DM and CAD nonetheless hyperlipidemia was significantly increased compared to controls.

We did not confirm UTI prevalence in patients compared to controls, but the limitation of self-reporting, might interfere, creating misinterpretations. Tonsillectomy was not associated with PBC. By contrast cholecystectomy was, in accordance with the French findings [4].

More patients than controls reported malignancies, one third of which were HCC, giving an OR 4.29 (95%CI 1.13-16.13). This is in agreement with the increased risk for malignancies found in 212 Greek patients previously reported [32].

Non significant difference in reproductive history of PBC patients was identified.

## Conclusions

In conclusion, this study has demonstrated that hyperlipidaemia and autoimmune diseases were significantly increased not only in PBC patients as expected, but also in their younger FDR compared to controls. Primary educational level, cholecystectomy and the presence of at least another autoimmune disease were found to be putative risk factors for PBC in our Greek population. The increased prevalence of malignancies previously reported was also confirmed in our study.

Given the high occurrence of familial PBC, the screening of PBC family members with AMA, especially those with at least another autoimmune disease, could be suggested in our population in order to diagnose and eventually treat the disease at an earlier stage.

## Abbreviations

PBC, primary biliary cirrhosis; FDR, first degree relatives; AOR, adjusted odds ratio; AMA, anti-mitichondrial antibodies; PDC, pyruvate dehydrogenase complex; E.coli, Escherichia coli; MMTV, mouse mammalian tumor virus; HRT, hormone replacement therapy; UTI, urinary tract infections; BMI, body mass index; HT, hypertension; DM, diabetes mellitus; CAD, coronary artery disease; COPD, chronic obstructive pulmonary disease; TPO, thyroperoxidase; TG, thyroglobulin; ALT, alanine aminotransferase; AST, aspartate aminotranferase; ALP, alkaline phosphatase; γ-GT, γ-glutamine transferase (γ-GT); HDL, high density lipoprotein; LDL, low density lipoprotein; RF, rheumatoid factor; ANA, antinuclear antibodies; SMA, anti-smooth muscle antibodies; SPSS, statistical package for social sciences; RA, rheumatoid arthritis; S.L.E., systemic lupus erythematosus; IBD, inflammatory bowel disease; HCC, hepatocellular carcinoma.

## Competing interests

The authors declare that they have no competing interests.

## Authors’ contributions

AM: performed patients’ and FDRs’ interviews, collected blood samples, reviewed patients’ medical files, participated in the statistical analysis, involved in literature research and contributed to writing of the manuscript. MK: participated in the design of the study, collected blood samples, involved in literature research and contributed to writing of the manuscript. GC: participated in the design of the study and performed the statistical analysis. JMEM: collected blood samples. AD: performed laboratory tests. MT: performed pathological analysis of liver samples. EK: conceived of the study, participated in its design and coordination, and wrote the manuscript. All authors read and approved the final manuscript.

## Pre-publication history

The pre-publication history for this paper can be accessed here:

http://www.biomedcentral.com/1471-230X/12/110/prepub

## References

[B1] Talwalkar JA Lindor KD Primary biliary cirrhosis Lancet 2003 362 53 61 10.1016/S0140-6736(03)13808-1 12853201

[B2] Poupon R Primary biliary cirrhosis: A 2010 update J Hepatol 2010 52 745 58 10.1016/j.jhep.2009.11.027 20347176

[B3] Selmi C Mayo MJ Bach N Ishibashi H Invernizzi P Gish RG Gordon SC Wright HI Zweiban B Podda M Gershwin ME Primary biliary cirrhosis in monozygotic and dizygotic twins: Genetics, epigenetics, and environment Gastroenterology 2004 127 2 485 92 10.1053/j.gastro.2004.05.005 15300581

[B4] Corpechot C Chritien Y Chazouillieres O Poupon R Demographic, lifestyle, medical and familial factors associated with primary biliary cirrhosis J Hepatol 2010 53 162 9 10.1016/j.jhep.2010.02.019 20471130

[B5] Lazaridis KN Juran BD Boe GM Slusser JP de Andrade M Homburger HA Ghosh K Dickson ER Lindor KD Petersen GM Increased prevalence of antimitochondrial antibodies in first-degree relatives of patients with primary biliary cirrhosis Hepatology 2007 46 785 92 10.1002/hep.21749 17680647

[B6] Bogdanos DP Smyk DS Rigopoulou EI Mytilinaiou MG Heneghan MA Selmi C Eric Gershwin M. Twin studies in autoimmune disease: Genetics, gender and environment J Autoimmun 2012 May 38 156 69 10.1016/j.jaut.2011.11.003 22177232

[B7] Watt FE James OFW Jones DEJ Patterns of autoimmunity in primary biliary cirrhosis patients and their families: a population-based cohort study Q J M 2004 97 397 406 10.1093/qjmed/hch078 15208427

[B8] Prince MI Chetwynd A Diggle P Jarner M Metcalf JV James OF The geographical distribution of primary biliary cirrhosis in a well defined cohort Hepatology 2001 34 1083 8 10.1053/jhep.2001.29760 11731995

[B9] McNally RJ Ducker S James OF Are transient environmental agents involved in the cause of primary biliary cirrhosis? Evidence from space-time clustering analysis Hepatology 2009 50 1169 74 10.1002/hep.23139 19711423

[B10] Ala A Stanca CM Bu-Ghanim M Ahmado I Branch AD Schiano TD Odin JA Bach N Increased prevalence of primary biliary cirrhosis near superfund toxic waste sites Hepatology 2006 43 525 31 10.1002/hep.21076 16496326

[B11] Jones DE Pathogenesis of primary biliary cirrhosis Gut 2007 56 1615 24 17641080 10.1136/gut.2007.122150 PMC2095651

[B12] Bogdanos DP Baum H Grasso A Okamoto M Butler P Ma Y Rigopoulou E Montalto P Davies ET Burroughs AK Vergani D Microbial mimics are major targets of cross-reactivity with human pyruvate dehydrogenase in primary biliary cirrhosis J Hepatol 2004 Jan 40 1 31 9 10.1016/S0168-8278(03)00501-4 14672611

[B13] Vilagut L Pares A Vinas O Vila J Jiménez de Anta MT, Rodés J. Antibodies to mycobacterial 65kd heat shock protein cross react with the main mitochondrial antigens in patients with primary biliary cirrhosis Eur J Clin Invest 1997 27 667 72 10.1046/j.1365-2362.1997.1690724.x 9279530

[B14] Klein R Wiebel M Engelhart S Berg PA Sera from patients with tuberculosis recognize the m2aepitope (e2subunit of pyruvate dehydrogenase) specific for primary biliary cirrhosis Clin Exp Immunol 1993 92 308 16 7683589 10.1111/j.1365-2249.1993.tb03397.x PMC1554802

[B15] Padgett KA Selmi C Kenny TP Leung PS Balkwill DL Ansari AA Coppel RL Gershwin ME Phylogenetic and immunological definition of four lipoylated proteins from novosphingobium aromaticivorans, implications for primary biliary cirrhosis J Autoimmun 2005 24 209 19 10.1016/j.jaut.2005.01.012 15848043

[B16] Mattner J Savage PB Leung P Oertelt SS Wang V Trivedi O Scanlon ST Pendem K Teyton L Hart J Ridgway WM Wicker LS Gershwin ME Bendelac A Liver autoimmunity triggered by microbial activation of natural killer T cells Cell host Microbe 2008 3 5 304 15 10.1016/j.chom.2008.03.009 18474357 PMC2453520

[B17] Bogdanos LD Pusl T Rust C Vergani D Beuers U Primary biliary cirrhosis following lactobacillus vaccination for recurrent vaginitis J Hepatol 2008 49 466 73 10.1016/j.jhep.2008.05.022 18644655

[B18] Leung PS Park O Matsumura S Ansari AA Coppel RL Gershwin ME Is there a relation between Chlamydia infection and primary biliary cirrhosis? Clin Dev Immunol 2003 10 227 33 10.1080/10446670310001642429 14768955 PMC2485416

[B19] Abdulkarim AS Petrovic LM Kim WR Angulo P Lloyd RV Lindor KD Primary biliary cirrhosis: An infectious disease caused by chlamydia pneumoniae? J of Hepatol 2004 40 380 84 10.1016/j.jhep.2003.11.033 15123349

[B20] Boomkens SY de Rave S Pot RG Egberink HF Penning LC Rothuizen J Zondervan PE Kusters JG The role of helicobacter spp in the pathogenesis of primary biliary cirrhosis and primary sclerosing cholangitis FEMS Immunol Med Microbiol 2005 44 221 25 10.1016/j.femsim.2004.11.002 15866219

[B21] Xu L Shen Z Guo L Fodera B Keogh A Joplin R O’Donnell B Aitken J Carman W Neuberger J Mason A Does a beta retrovirus infection trigger primary biliary cirrhosis? Proc Nat Ac Sci USA 2003 100 8454 9 10.1073/pnas.1433063100 PMC166250 12832623

[B22] Mason A Xu L Shen Z Fodera B Joplin R Neuberger J O’Donnell B Patients with primary biliary cirrhosis make antiviral and anti-mitochondrial antibodies to mouse mammary tumor virus Gastroenterology 2004 127 6 1863 4 10.1053/j.gastro.2004.10.024 15578536

[B23] Long A Quan C Van de Water J Nantz MH Kurth MJ Barsky D Colvin ME Lam KS Coppel RL Ansari A Gershwin ME Immunoreactivity of organic mimeotopes of the e2 component of pyruvate dehydrogenase: Connecting xenobiotics with primary biliary cirrhosis J Immunol 2001 167 2956 63 11509645 10.4049/jimmunol.167.5.2956

[B24] Rieger R Gershwin ME The x and why of xenobiotics in primary biliary cirrhosis J Autoimmun 2007 28 76 84 10.1016/j.jaut.2007.02.003 17360156 PMC1934549

[B25] Prince MI Ducker SJ James OF Case–control studies of risk factors for primary biliary cirrhosis in two United Kingdom populations Gut 2010 59 4 508 12 10.1136/gut.2009.184218 20332522

[B26] Gershwin ME Selmi C Worman HJ Gold EB Watnik M Utts J Lindor KD Kaplan MM Vierling JM USA PBC Epidemiology Group. Risk factors and comorbidities in primary biliary cirrhosis: a controlled interview-based study of 1032 patients Hepatology 2005 42 5 1194 202 10.1002/hep.20907 16250040 PMC3150736

[B27] Parikh-Patel A Gold EB Worman H Krivy KE Gershwin ME Risk factors for primary biliary cirrhosis in a cohort of patients from the United States Hepatology 2001 33 16 21 10.1053/jhep.2001.21165 11124815

[B28] Tsuji K Watanabe Y Van De Water J Familial primary biliary cirrhosis in Hiroshima J Autoimmun 1999 13 171 8 10.1006/jaut.1999.0299 10441183

[B29] Su TC Hwang JJ Kao JH Hypercholesterolemia in primary biliary cirrhosis N Engl J Med 2007 357 1561 62 10.1056/NEJMc071467 17928612

[B30] Dudnik LB Azyzova OA Solovyova NP Savchenkova AP Pokrovskaya MA Primary biliary cirrhosis and coronary atherosclerosis: protective antioxidant effect of bilirubin Bull Exp Biol Med 2008 145 18 22 10.1007/s10517-008-0019-4 19023993

[B31] Floreani A Variola A Niro G Plasma adiponectin levels in primary biliary cirrhosis: a novel perspective for link between hypercholesterolemia and protection against atherosclerosis Am J Gastroenterol 2008 103 1959 65 10.1111/j.1572-0241.2008.01888.x 18564121

[B32] Deutsch M Papatheodoridis GV Tzakou A Hadziyannis SJ Risk of hepatocellular carcinoma and extrahepatic malignancies in primary biliary cirrhosis Eur J Gastroenterol Hepatol 2008 20 5 10.1097/MEG.0b013e3282f163ed 18090982

